# Deciphering the morpho-physiological and biochemical response of sunflower hybrids with the application of biochar and slow-release nitrogen fertilizers under drought stress for sustainable crop production

**DOI:** 10.3389/fpls.2025.1541123

**Published:** 2025-03-04

**Authors:** Shabir Hussain, Mehrab Khan, Muhammad Tanveer Altaf, Muhammad Nadeem Shah, Alanoud T. Alfagham

**Affiliations:** ^1^ Institute of Agronomy, Bahauddin Zakariya University, Multan, Punjab, Pakistan; ^2^ Department of Field Crops, Faculty of Agriculture, Recep Tayyip Erdoğan Üniversity, Pazar, Rize, Türkiye; ^3^ Department of Agriculture, Government College University, Lahore, Punjab, Pakistan; ^4^ North Florida Research and Education Centre (NFREC), University of Florida, Quincy, FL, United States; ^5^ Department of Botany and Microbiology, College of Science, King Saud University, Riyadh, Saudi Arabia

**Keywords:** sustainable agriculture, biochar, drought stress, slow-release nitrogen fertilizers, antioxidants, reactive oxygen species, sunflower productivity

## Abstract

Agriculture problems like drought stress and improper fertilization like overuse of nitrogen fertilizers for maximum productivity are the problem responsible for low yield of crop and environmental pollution. Biochar and slow releasing nitrogen fertilizers (SRNF) application in agriculture are the sustainable practices being used for better crop nutrient management strategies, since the well-recognized environmental problem caused by overusing fertilizers. Biochar also used as tools for sustainable way alleviating drought stress. For this, two-year field study was planned with randomized complete block designed (RCBD) and was replicated three time. Treatments included the two irrigation conditions like normal irrigation (CK) and drought stress (DS), two biochar treatments like biochar (BC) and without biochar (WBC); and three application of SRNF like zinc-coated urea (ZCU), sulfur-coated urea (SCU) and non-coated simple urea (SU). Results revealed that drought stress significantly reduced plant height (20.7%), stem diameter (25.6%), and achene yield (25.9%), while increasing antioxidant activity. Biochar mitigated these effects, increasing plant height by 23.2% and achene yield by 12.0% under drought stress. Among SRNFs, ZCU was most effective, improving photosynthetic rate (18.5%), chlorophyll content (12.3%), and achene yield (19.6%) under drought conditions. The combination of biochar and ZCU improved soil health, water retention, and nutrient efficiency, leading to enhanced plant growth and yield. Statistical analysis confirmed significant differences among treatments.

## Introduction

Worldwide, sunflowers (*Helianthus annuus* L.) are cultivated on around 24.77 million hectares, making it one of the most important oilseed crops. Seeds from this crop contain 25–48% oil and 20–27% protein, making it an important crop for human nutrition and industrial use in the production of edible oil. Sunflower output in Pakistan is still below its potential, with average yields falling well short of international standards. Therefore, the country’s need for vegetable oil is met primarily through imports. Drought stress is a major issue that reduces agricultural output, but there are other causes, including biotic and abiotic pressures, that contribute to these yield gaps (Ebrahimian et al., 2019). Sustainable crop production strategies are becoming more important as climate change makes droughts more common.

As a significant abiotic factor, drought stress hinders crop development and productivity by interfering with a number of biochemical and physiological processes. Photosynthetic efficiency and harvest yield are both diminished as a result of its effect on chlorophyll synthesis (Sunaina et al., 2019; Ma et al., 2022). Hydrogen peroxide and superoxide anions are reactive oxygen species (ROS) that plants produce in excess during drought, and they damage plant cells through oxidative stress. Protein synthesis, enzyme activity, and membrane stability are all negatively impacted by this oxidative stress, which in turn hinders plant growth and harvest potential (Cui et al., 2017; Vijayaraghavareddy et al., 2022). How much harm a drought does depends on how long it lasts, what kinds of plants it affects, and how severe the drought is. In order to guarantee food security and satisfy the increasing demands of the world’s population, it is essential to address the issues caused by drought (Agarwal et al., 2016).

Agricultural production is greatly enhanced by nitrogen fertilizers; yet, the overuse and lack of regulation of this fertilizer leads to inefficient nutrient usage and environmental damage. Soil loss and groundwater pollution are two consequences of nitrogen application; studies show that as much as 50% of applied nitrogen is lost through processes such as volatilization, leaching, and runoff (Rahman et al., 2018; Hochman and Horan, 2018). Not only do these losses drive up production costs, but they also decrease the efficiency of fertilizer. By releasing nutrients gradually, slow-release nitrogen fertilizers (SRNFs) improve crop output, reduce environmental impact, and increase nitrogen usage efficiency (Naz and Sulaiman, 2016; Zhang et al., 2015). One kind of SRNF that has proven to be quite useful in maintaining nutrient availability and bolstering plant development and productivity is urea coated with sulfur or zinc.

More and more, biochar a carbon-dense substance made from the pyrolysis of organic matter is being acknowledged as a long-term solution for enhancing the condition of soil. This resource is highly beneficial for reducing the impact of abiotic conditions, such as drought, because it improves soil structure, water retention, and nutrient availability (Shah et al., 2023; Tang et al., 2022). Biochar makes plants more drought-resistant by increasing their chlorophyll production, stomatal conductance, and antioxidant enzyme activity (Paneque et al., 2016; Ramzani et al., 2017). Further aiding crop performance in challenging environments, biochar decreases soil compaction, increases water penetration, and promotes microbial activity (Haider et al., 2020). A synergistic approach to nitrogen management and drought stress reduction can be achieved through the combination of biochar with SRNFs, leading to improved crop yield and sustainability.

The combined effects of biochar and slow-release nitrogen fertilizers (SRNFs) on sunflower hybrids under drought stress remain understudied. While biochar enhances soil health and water retention and SRNFs ensure gradual nutrient release, their integration could be a promising strategy for improving sunflower growth in low-water conditions. This study investigates the impact of this combination on the growth, physiology, and biochemistry of sunflower hybrids, aiming to develop sustainable farming practices for drought-prone regions. In order to find long-term solutions for growing sunflowers in areas with limited water, this study aims to evaluate the effects of these additives on crop development, yield, and drought resistance. The results of this study should pave the way for more sustainable integrated agronomic approaches that guarantee great yields with little impact on the environment.

## Materials and methods

### Experimental site, treatments and study design

The research was conducted at the Institute of Agronomy, Bahauddin Zakariya University, Multan, Pakistan, over a two-year period from 2021 to 2022 and 2022 to 2023. The experiment employed two biochar (with and without) applications (BC and WBC) in conjunction with three types of slow-release nitrogen fertilizer: zinc-coated urea (ZCU), sulfur-coated urea (SCU), and non-coated simple urea (SU). Furthermore, two irrigation regimens were implemented: normal irrigation (CK) and drought stress (DS). This study implemented a split-split plot layout and implemented a randomized complete block design (RCBD) with three replications. The tertiary plots were subjected to SRNF treatments, the secondary plots received biochar treatments, and the primary plots were allocated irrigation regimes.

### Nitrogen fertilizers composition

There were three different types of coated urea: plain urea (SU) that did not contain any coating, zinc-coated urea (ZCU), which had 32% nitrogen and 1% zinc, and sulfur-coated urea (SCU), which contained 32% nitrogen and 5% sulfur. Following the treatment plan, a total of 60 kg of nitrogen per acre was applied as fertilizer. The total amount of nitrogen was applied in three stages: half at sowing, 25% during the second irrigation, and the last quarter during flowering.

### Biochar preparation and application

Cotton sticks were sun-dried, crumbled, and pyrolyzed at 450°C for two hours to create biochar. Biochar was made by first sun-drying cotton sticks, then chopping them into little pieces, and last pyrolyzing them at 450°C for two hours., according to the procedure described by Qayyum et al. (2021). Soil stabilization was achieved by applying and mixing it at a rate of 10 tons per hectare one month before planting. Its porous structure and high water-holding capacity improve soil moisture retention and nutrient efficiency. Applied at 10 tons per hectare, it reduces drought stress by enhancing water availability. The physiochemical analysis of biochar was done, it contains 23% organic carbon, while 61.5% dry matter, 1.11% nitrogen, 0.45mg kg^-1^ phosphorus, and 0.61 mg kg^-1^ potassium.

### Experimental soil and environment

**Figure 1:**
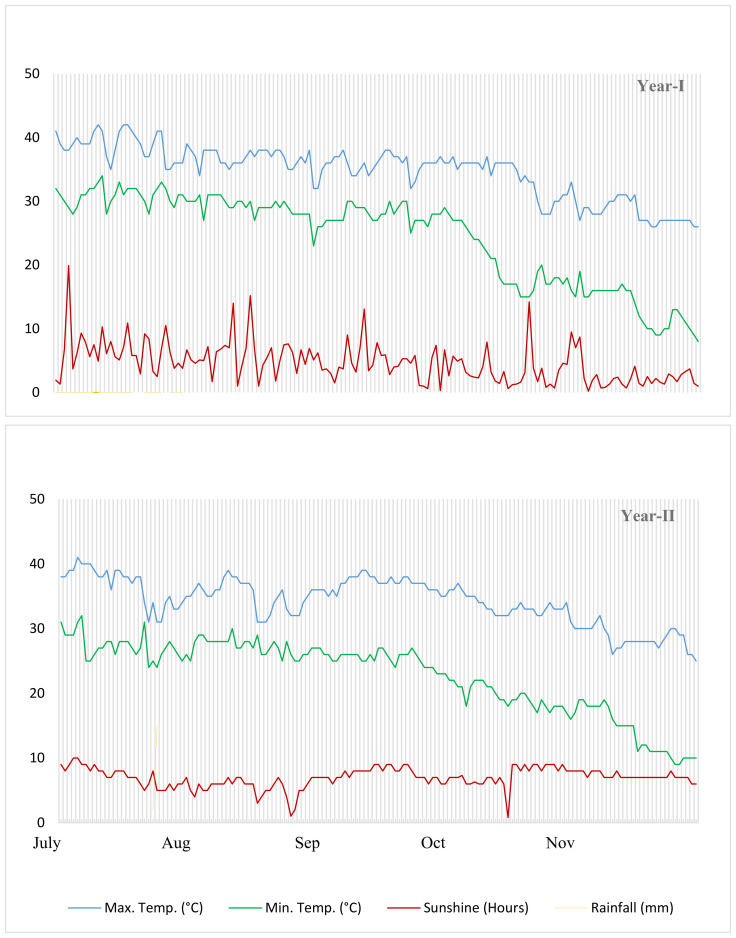
Climate conditions.

The fertility level of the experimental soil was evaluated using a physico-chemical examination prior to seeding. At a depth of 15 cm, soil samples weighing 100 g each were taken at random. There was a 24-hour oven drying period at 105°C after the samples were air-dried and sieved to eliminate debris. To find the soil’s dry weight, a computerized weighing balance was employed. The sandy loam soil had the following characteristics: a pH of 7.5, an EC of 2.6 dS m^-^¹, and an organic matter content of 1.02 percent. Its texture was also recognized. Potassium, 9.50 parts per million (ppm), total available nitrogen (0.05 percent), and phosphorus (120 ppm) were the measured concentrations. The environmental condition of the experiment is given in (**Figure 1**).

### Field capacity management

In order to keep the soil’s field capacity constant, its moisture levels were measured on a frequent basis throughout the experiment. For precise irrigation management, an XLUX^®^ T10 Soil Moisture Meter was utilized. Consistent maintenance of the required moisture levels was ensured by applying irrigation as needed using a cut-throat flume.

### Crop husbandry

To achieve the best possible tilth, increased aeration, and a fine seedbed that would be ideal for planting crops, the soil was rotavated once and plowed three times. A north-south orientation was used to prepare the beds, and seeds were scattered at a 9 cm spacing at the very top of each bed by hand. At a rate of 2 g kg^-1^, the seeds were treated with Thiophanate Methyl to prevent the occurrence of illness. The application rate of Pendimethalin (33 percent EC) was 1,000 mL per acre for pre-emergence weed suppression.

After the crop reached the V4 growth stage, the experimental strategy called for the use of drought stress treatments. In accordance with the guidelines of the Punjab Agriculture Department, 40 kg of phosphorus and 25 kg of potassium fertilizer were sprayed per hectare, respectively. At the time of planting, the soil was amended with basal doses of triple superphosphate (20 percent P) and muriate of potash (60 percent KCl). Also implemented were agronomic practices including thinning to reduce intra-plant competition and gap filling to keep plant populations at an ideal level. The other methods of crop protection were consistently used in every treatment.

### Data collection

Five plants randomly from each experimental unit were tagged for data collection.

### Morphological parameters

Morphological parameters were measured as par standard procedure.

### Gas exchange parameters

Analytical Development Company of Hoddesdon, England, manufactured an infrared gas analyzer (IRGA) that was used for measuring photosynthesis, transpiration, and stomatal conductance. A SPAD meter was used to measure the chlorophyll concentration (SPAD-502 Chlorophyll Index, SCI).

### Fatty acid profile

In line with the method developed by Chen et al. (2008), the fatty acid composition of stearic acid, palmitic acid, linolenic acid and oleic acid was analyzed with the aid of near-infrared spectroscopy (NIRS).

### Anitioxidant enzyme activities

For purposes of antioxidant enzyme activity profiling, 0.3 g of fresh leaf samples was homogenized in 3 mL of cold 50 mM sodium phosphate buffer (pH 7.8). The homogenate was then centrifuged in a centrifuge for 20 minutes for 4°C at 15,000 rpm. Enzyme activity experiments were conducted using the recovered supernatant, as explained below.

Superoxide dismutase activity was measured using the method by Giannopolitis and Ries (1977). This method is based on the enzyme’s ability to prevent the photoreduction of nitroblue tetrazolium (NBT). The enzyme activity was expressed in micromoles per minute per milligram of protein. To perform the reaction, a mixture was prepared using sodium phosphate buffer (50 mM, pH 7.8), riboflavin (1.3 mM), methionine (13.5 mM), EDTA (75 mM), and NBT (50 mM). Specifically, the mixture included 1 milliliter each of NBT and riboflavin, 500 microliters each of methionine and EDTA, 950 microliters of sodium phosphate buffer, and 50 microliters of the enzyme solution. The reaction was initiated by exposing the samples to light from fifteen fluorescent lamps (intensity of 78 mmol m^-^² s^-^¹) for 15 minutes.

Peroxidase (POD) and catalase (CAT) activity was assessed using the methodology developed by Chance and Maehly (1955) with some amendments. Reaction mixture containing of 900 µL of 5.9 mM H_2_O_2_, 2 mL of 50 mM sodium phosphate buffer with 7.8 pH and 100 µL of supernatant used for CAT activity assessment. At intervals of 30 seconds over a period of 5 minutes, changes in absorbance of the reaction mixture were observed at 240 nm wavelength using a UV-1900 spectrophotometer. During the absorption process, the UV lamp was turned on. The reaction mixture consisted of 2 mL of 500 µL of 40 mM H_2_O_2_, 50 mM sodium phosphate buffer having 7.8 pH, 400 µL of 20 mM guaiacol and 100 µL of supernatant used for assessing POD activity. Change in the absorbance of the reaction mixture was observed at a wavelength of 470 nm every 20 seconds during a period of 5 minutes. The activities of these enzymes were individually quantified based on the amount of protein present.

### Hydrogen peroxide

The concentrations of H_2_O_2_, as micromoles per gram of fresh leaves were determined by grinding the leaves under liquid nitrogen. Twenty powdered eucalyptus leaves weighed 300 mg which when treated with 3 mL of 0.1 percent w/v trichloroacetic acid (TCA) were centrifuged at 12000 rpm for 15 minutes. Following the procedure described by Velikova et al. (2000), the levels of H_2_O_2_ in the analyzed leaf samples were estimated. In the given reaction mixture the supernatant was 500 µL, potassium iodide was 1 mL of 1M while the potassium phosphate buffer, 10 mM at pH 7.0. For the determination of H_2_O_2_ concentrations, the absorbance of the resulting mixture was measured using a UV-1900 spectrophotometer from BMS, Canada at 390 nm.

### Ascorbate peroxidase

Measurement of activity of ascorbate peroxidase (APX) was done using the method described by Poiroux-Gonord et al. (2013). The enzyme’s activity was expressed in micromoles of substrate converted per milligram of protein per minute. The reaction mixture contained 13.42 µL of 2 mM H_2_O_2_, 100 µL of 50 mM potassium phosphate buffer (pH 7.0), 1 mL of 0.33 mM L-ascorbate and 100 µL of the enzyme supernatant. The absorbance at 290 nm was then obtained using a UV-1900 UV spectrophotometer of BMS, Canada after transferring the reaction mixture to a 1 mL quartz cuvette with a UV lamp. APX activity was determined at 30 seconds interval for a total of 180 seconds.

### Malondialdehyde (µmol g^-1^ FW)

Determination of MDA in the leaves was done using the method developed by Heath and Packer (1968) and expressed as µmol g^-^¹ FW. In preparation of the reaction mixture, one mL of the leaf extract was dipped in 0.5 percent TCA containing 1 mL of a 0.5 percent (w/v) TBA. The mixture was then incubated at 56°C at room temperature for half an hour before undergoing heat inactivation through a water bath run at 100°C for 5 minutes followed by an immediate transfer to an ice bucket. The samples were allowed to equilibrate to the indicated temperature before being centrifuged at 3,000 rpm for 10 minutes to allow sedimentation of the supernetant. A spectrophotometer was used to take absorbance readings at two different wavelengths: 532 nm and 600 nm. Absorbance values obtained were then used to calculate the MDA content through the equation given in the protocol.


$$\text{MDA level}(\mu \text{mol }{\text{g}}^{-1}\text{ FW})=\frac{\left(\text{A }532\text{nm}-\text{A }600\text{nm}\right)}{1.56}\times 105$$

### Total soluble protein (mg g^-1^ FW)

The total soluble protein (TSP) was estimated using the Lowry method (1951). Fresh leaves (0.5 g) were dissected in 1 mL of PBS at pH 7.2. The PBS contained 2.7 mM K^+^, 1 mM K^+^ dihydrogen phosphate, 10 mM Na^+^, and 1.3 mM Cl^-^. After homogenization, the sample was centrifuged at 10,000 rpm for 5 minutes to obtain the supernatant. To measure protein, the reaction mixture included 20 µL of cosmic blue dye, 780 µL of deionized water, and 20 µL of the plant extract. The absorbance of the oxidized protein was measured at 595 nm using a spectrophotometer (UV-1900, BMS, Canada), and the TSP concentration was calculated based on the absorbance values.


$$\text{Total soluble protein content}({\text{mg g}}^{-1}\text{FW})=\text{Absorption}+0.25\times (\frac{1}{\text{g sample}})$$

### Statistical analysis

For a comparison of the treatment effects, a two-way analysis of variance (ANOVA) with general linear models was used. The differences between the treatments in equal means comparison by Tukey’s test at 5% significance level were also highly significant. In the study statutory analysis, the software “Statistix 8.1” was employed to conduct all the tests. In each treatment, three replicates per genotype and a total of five plants per genotype were grown. All the data presented in the data visualizations was created with the assistance of the Microsoft Office 2016.

## Results

### Morphological parameters

The analysis of variance (ANOVA) is given in **Table 1** for the application of biochar and SRNFs significantly influenced most observed traits of sunflower under drought stress, indicating their effectiveness in improving plant growth and yield. Individual effects of irrigation and biochar treatments were highly significant, highlighting their critical role in mitigating drought impacts. Interactions between irrigation and biochar showed varying levels of significance, suggesting that their combined application enhances certain traits. However, some interactions, particularly those involving all factors, were not significant for all traits, indicating that the combined effects may depend on specific conditions or parameters. Overall, these findings demonstrate the potential of integrating biochar and slow-release nitrogen fertilizers to enhance sunflower performance under drought stress.

**Table 1:** Level of significant (p-value) effected by the application of biochar and slow release nitrogen fertilizers under drought stress on sunflower.

Observations		Plant height (cm)		Stem diameter (cm)		Head diameter (cm)		No. of leaves per plant		Number of achenes per head		1000 achene weight (g)		Achene yield (kg ha^-1^)		Biological yield (kgha^-1^)	
SOV	df	Year-I	Year-II	Year-I	Year-II	Year-I	Year-II	Year-I	Year-II	Year-I	Year-II	Year-I	Year-II	Year-I	Year-II	Year-I	Year-II
I	1	0.0002**	0.0003**	0.0011**	0.0006**	0.0022**	0.0022**	0.0015**	0.0011**	0.0015**	0.0021**	0.0008**	0.0009**	0.0011**	0.0008**	0.0007**	0.0006**
N	2	0.0000**	0.0000**	0.0000**	0.0000**	0.0000**	0.0000**	0.0000**	0.0000**	0.0000**	0.0000**	0.0000**	0.0000**	0.0000**	0.0000**	0.0000**	0.0000**
I×N	2	0.9050ns	0.0542ns	0.1773ns	0.0018**	0.1569ns	0.1751ns	0.8976ns	0.3781ns	0.7568ns	0.4142ns	0.3484ns	0.3548ns	0.1131ns	0.1046ns	0.2466ns	0.2151ns
B	1	0.0002**	0.0001**	0.0000**	0.0000**	0.0000**	0.0000**	0.0000**	0.0000**	0.3757ns	0.0612ns	0.0000**	0.0000**	0.0034**	0.0005**	0.0007**	0.3297ns
I×B	1	0.1062ns	0.0472*	0.7595ns	0.2641ns	0.0006**	0.0017**	0.0033**	0.0147*	0.0315*	0.0147*	0.0544ns	0.0024**	0.0040**	0.0327*	0.0847ns	0.7651ns
N×B	2	0.1624ns	0.0991ns	0.6926ns	0.1118ns	0.0672ns	0.0697ns	0.1819ns	0.5347ns	0.3074ns	0.2934ns	0.2522ns	0.9697ns	0.1521ns	0.1622ns	0.5852ns	0.2417ns
I×N×B	2	0.0499*	0.2491ns	0.0486*	0.0232*	0.0033**	0.0029**	0.0110*	0.0345*	0.0086**	0.0200*	0.0001**	0.0000**	0.0819ns	0.0623ns	0.0234*	0.1592ns

SOV, Source of variance; df, Degree of freedom; I, Irrigation regimes; N, Slow releasing nitrogen fertilizer; B, Biochar treatments. P=0.05%.

*, significant; **, highly significant.

**Figure 2:**
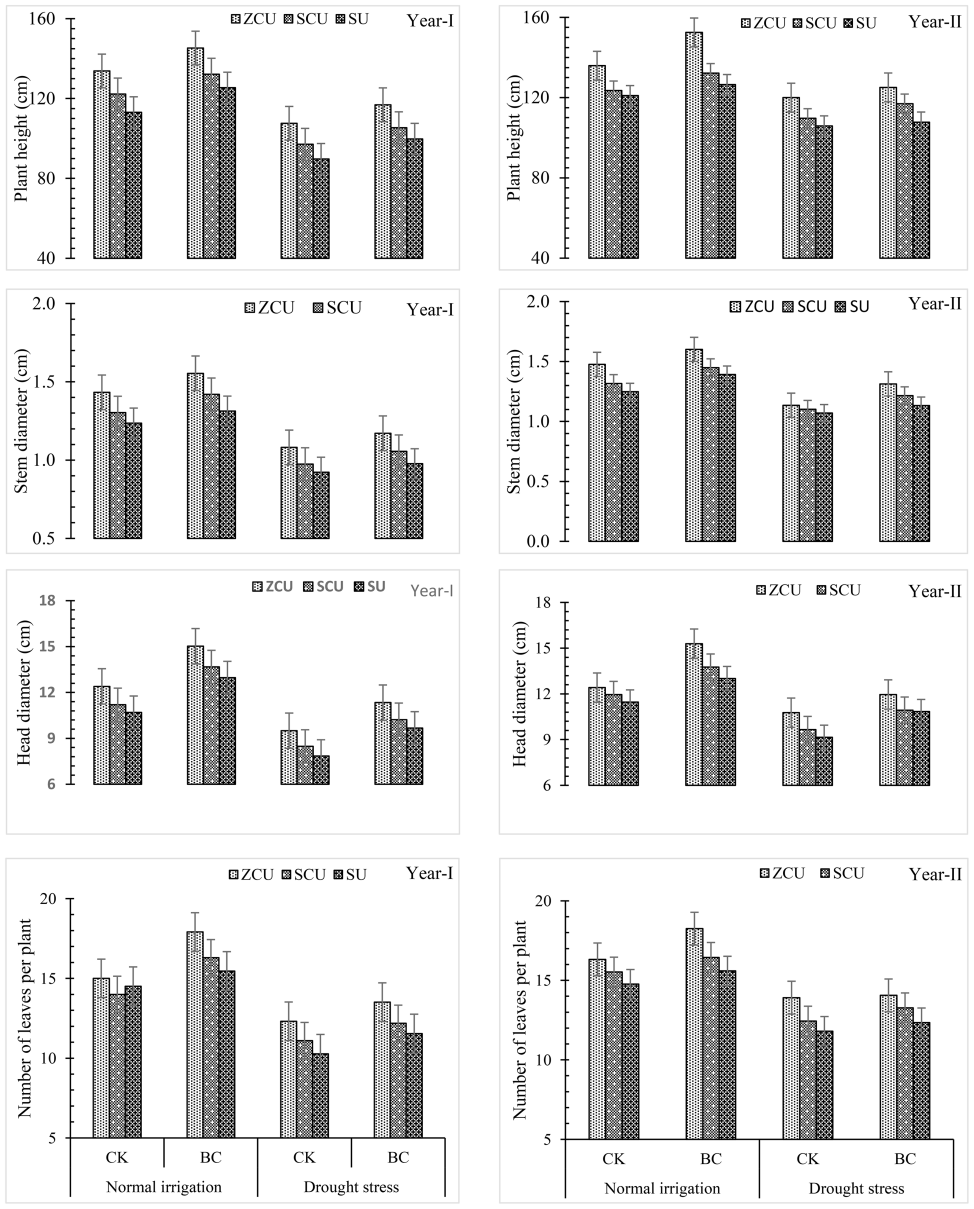
Plant growth parameters like plant height (cm), stem diameter (cm), number of leaves per plant and head diameter (cm) effected by slow release nitrogen fertilizers and biochar under drought stress.

Phenotypic characters namely plant height, stem diameter, number of tillers, and head diameter were found influential positively under the influence of biochar, irrigation treatments and slow-release nitrogen fertilizers analyzed through growth parameters during the two years study (**Figure 2**).

From growth promotion under normal irrigation and drought stress conditions, ZCU was the most effective of the three evaluated SRNF types followed by SCU and SU. Generally, application of ZCU raised plant height by 20.6 and 20.0%, head diameter by 18.4 and 19.9%, and leaf per plant by 17.0 and 21.2% under normal irrigation and drought stress conditions in comparison with the lowest yield shown by SU. Finally, it was established that there was a significant relationship between the enhancement in the yield of the product and the other yield influencing factors. As indicated by the following data obtained under normal irrigation and drought stress; ZCU raised achenes per head by 17.5 percent and 19.0 percent; 1000 achene weight by 17.0 percent and 20.1 percent; achene yield by 18.9 percent and 19.6 percent; and biological yield 18.4 percent and 20.9 percent. Results emerging from this study indicate that ZCU is a superior method of irrigation treatments for accelerating plant growth and yield enhancement compared to other methods under all the studied conditions.

The stunted growth, yield and other yield attributes characters due to decrease in irrigation water was recorded when the plants were irrigated under drought stress conditions than normal irrigation conditions. The overall plant height, stem diameter, head diameter and total number of leaves decreased significantly when plants experienced drought stress by 20.7%, 25.6%, 26.7% and 29.2% respectively. Consequently, reducing head size led to a 25.6% reduction in the number of achenes per head, 26.1% reduction in weight of 1000 achenes, 25.9% reduction in achene yield and a decrease of 27.1% in biological yield. Overall, the following results show the effects of drought stress on sunflower yields.

**Figure 3:**
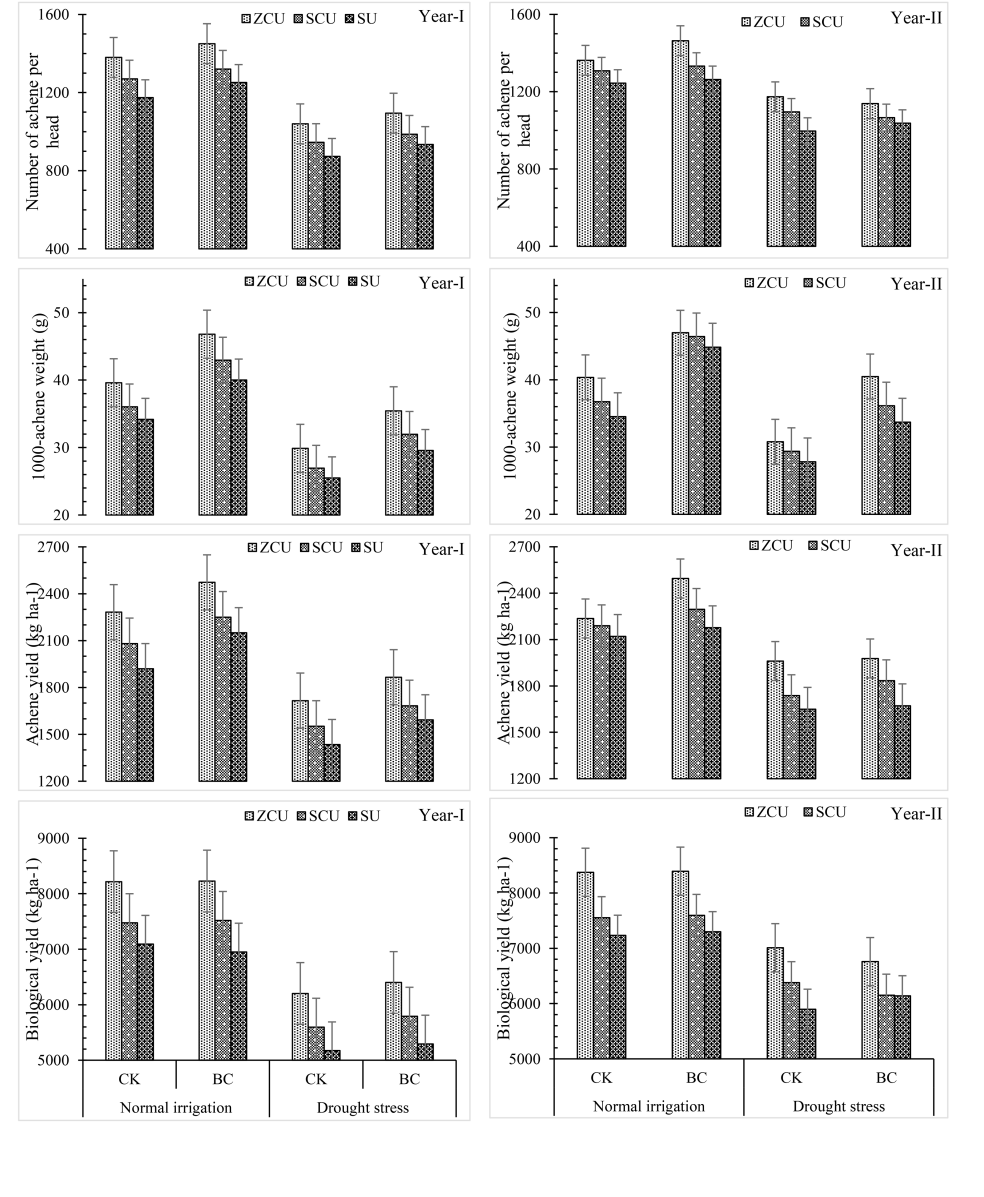
Yield related parameters like number of achene per head, 1000-achene weight (g), and achene by slow release nitrogen fertilizers and biochar under drought stress.

Incorporation of biochar led to mitigation of the deleterious impacts of drought stress as well as enhanced growth and yield parameters. In normal irrigation conditions and drought scenarios, plant height, stem diameter, head diameter and number of leaves were enhanced by 12.2%, 11.3%, 15.7% and 23.2%, 23.4% and increased the number of leaves by 19.4% and 12.3% respectively, when treated with biochar. It also enhanced yield related characteristics to a higher degree of efficiency in achenes per head, 1000-achene weight, achene yield, and biological yield by 7.5% and 6.9%, 29.9% and 31.5%, 12.0% and 11.0%, 0.90 and 4.13% respectively under normal irrigation and drought stress. It becomes clear from the study findings that the use of biochar can enhance yields and growth of crops for short irrigation (**Figure 3**).

### Gas exchange parameters and chlorophyll contents

**Figure 4:**
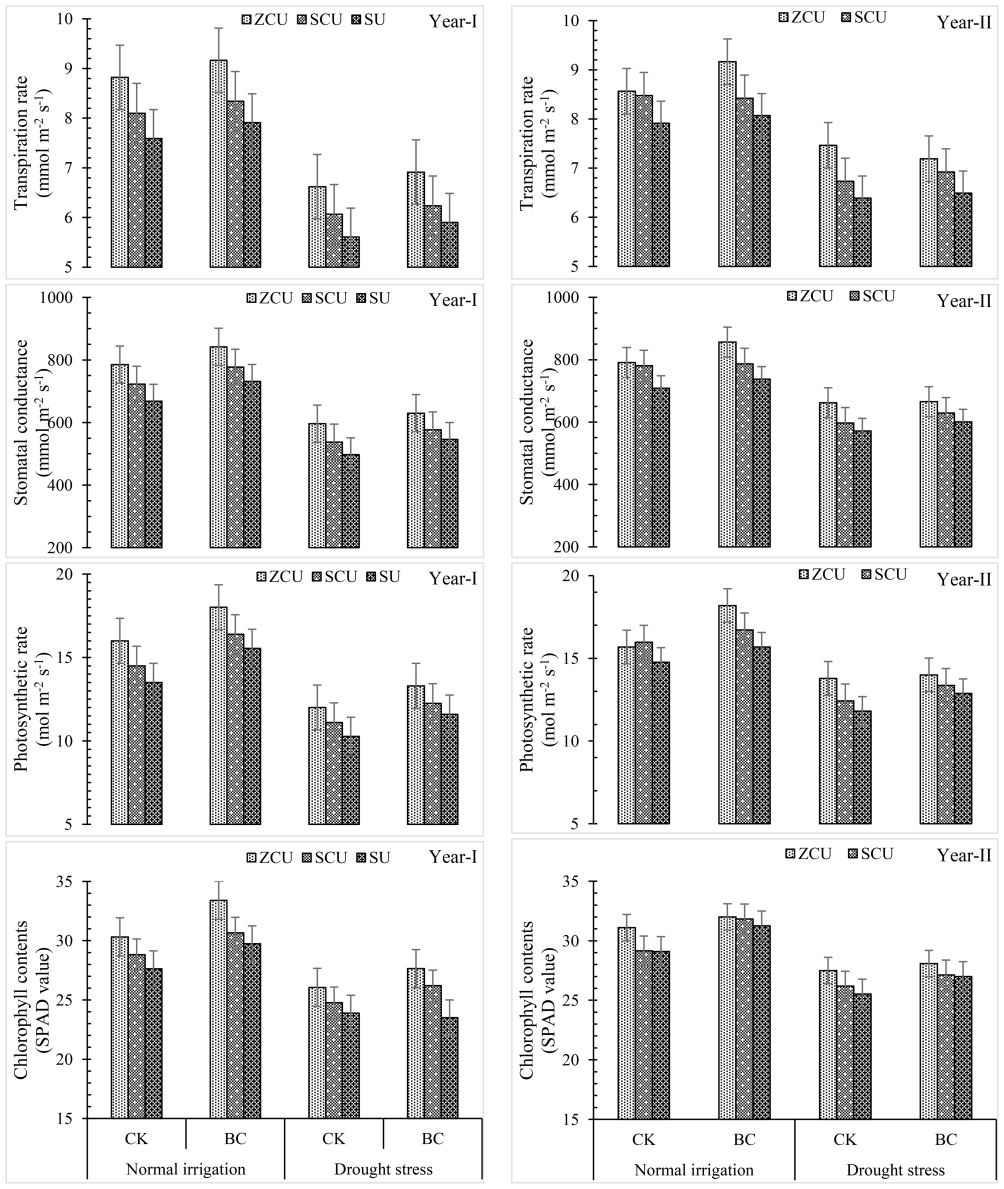
Crop physiological parameters like transpiration rate, stomatal conductance, photosynthetic rate and chlorophyll contents (SPAD value) effected by slow release nitrogen fertilizers and biochar under drought stress.

Transpiration rate, stomatal conductance, photosynthetic rate, and chlorophyll content were among the gas exchange parameters that were considerably impacted by the use of slow-release nitrogen fertilizers (SRNF), various irrigation regimes, and biochar (BC) treatments (**Figure 4**). Similar patterns to those seen for morphological characteristics of crops were also seen for these physiological variables. The gas exchange parameters and chlorophyll content were highest for zinc-coated urea (ZCU), sulfur-coated urea (SCU), and non-coated simple urea (SU) among the SRNF treatments. The results showed that zinc-coated urea performed better than SU treatment in several respects, including transpiration rate (16.2% increase), stomatal conductance (19.9% increase), photosynthetic rate (18.5% increase), and chlorophyll content (12.3%) under normal irrigation and drought stress, respectively. These findings show that ZCU is effective in improving physiological traits under different irrigation conditions.

Results from an examination of irrigation schedules showed that, in comparison to regular irrigation, drought stress drastically decreased gas exchange parameters and chlorophyll content. When plants are in a drought, their transpiration rate drops by 26.1%, their stomatal conductance drops by 25.8%, their photosynthetic rate drops by 26.2%, and their chlorophyll content drops by 17.2%.

Nonetheless, gas exchange parameters and chlorophyll content were enhanced by applying biochar under both irrigation regimes. In normal irrigation and drought stress conditions, respectively, the application of biochar resulted in a 7.02% and 5.28% increase in transpiration rate, a 9.49% and 9.82% boost in stomatal conductance, a 16.0 and 13.1 percent increase in photosynthetic rate, and an 11.14% and 6.06% increase in chlorophyll content. These results demonstrate that biochar can improve plant physiological performance in drought.

### Fatty acid profile

**Figure 5:**
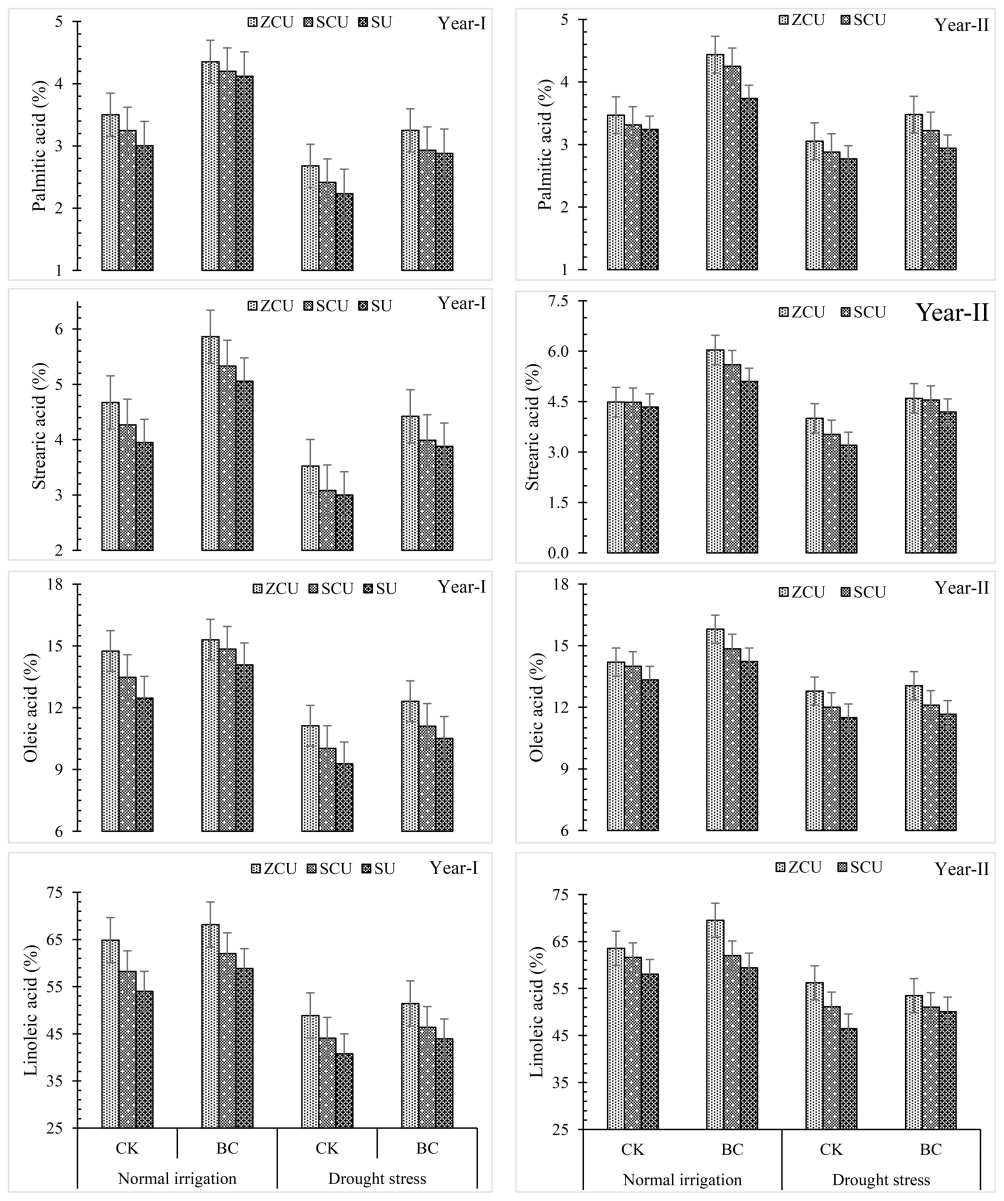
Fatty acid profile like palmitic acid, stearic acid, oleic acid and linoleic acid effected by slow release nitrogen fertilizers and biochar under drought stress.

Biochar, irrigation schedules, and slow-release nitrogen fertilizers (SRNF) had a substantial impact on the fatty acid composition of sunflower seeds (**Figure 5**). The treatments that produced the highest levels of fatty acids were zinc-coated urea (ZCU) and the treatments that produced the lowest levels were non-coated simple urea (SU). In comparison to SU, ZCU increased the levels of palmitic acid by 18.8% and 19.9% under normal irrigation conditions and stearic acid by 18.4% and 25% under drought stress conditions, oleic acid by 16.1% and 19.9%, and linoleic acid by 20.1% and 21.0% under these same conditions.

When comparing levels of fatty acids during normal irrigation to those during drought stress, a clear trend emerged. The following acids showed reductions under drought stress: palmitic acid (25.6% reduction), stearic acid (27.9% reduction), oleic acid (27.0% reduction), and linoleic acid (24.6% reduction).

Fatty acid levels were found to be improved under both irrigation conditions when biochar was applied. Biochar increased the levels of palmitic acid by 37.2%, stearic acid by 34.6%, and oleic acid by 11.3%, and linoleic acid by 9.40% when grown under conventional irrigation conditions. Biochar increased levels of palmitic acid by 28.9%, stearic acid by 29.5%, and oleic acid by 10.7%, and linoleic acid by 7.71% when subjected to drought stress. These findings demonstrate that ZCU and biochar can improve the fatty acid profile of sunflower seeds, regardless of the presence or absence of water.

### Biochemical analysis

#### Antioxidant activity

**Figure 6:**
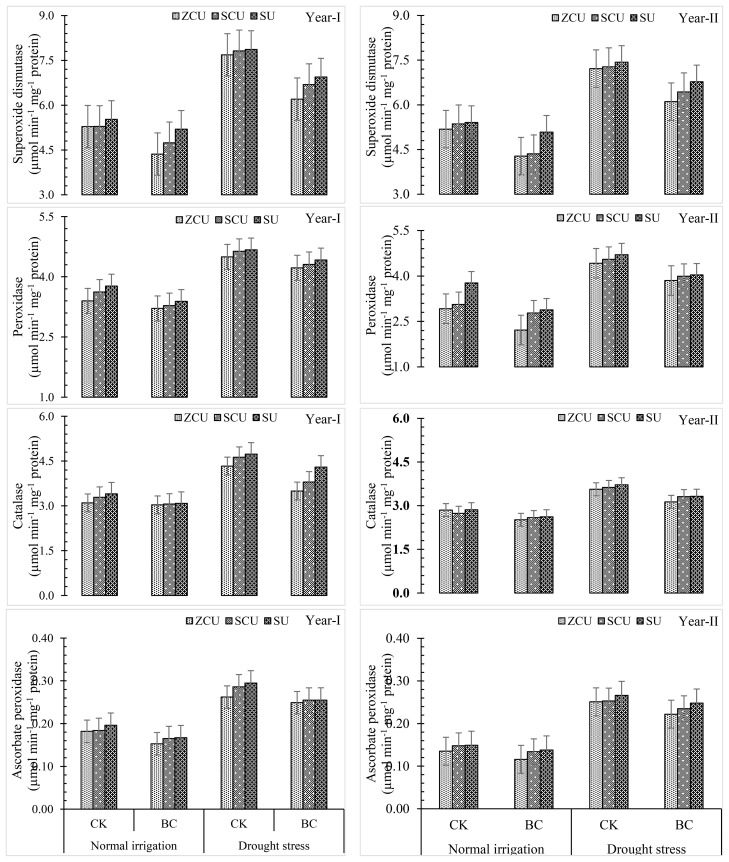
Antioxidant activity like Superoxide dismutase (SOD), peroxidase (POX), catalase (CAT) and ascorbate peroxidase (APX) effected by slow release nitrogen fertilizers and biochar under drought stress.

Application of biochar, changes in irrigation patterns, and slow-release nitrogen fertilizers (SRNF) had a substantial impact on antioxidant activities, including those of superoxide dismutase (SOD), peroxidase (POD), catalase (CAT), and ascorbate peroxidase (APX). Drought stress was associated with the greatest SOD, POD, CAT, and APX activities. Under both irrigation conditions, the antioxidant activity was further affected by the addition of biochar. When compared to normal irrigation, the activities of SOD, POD, CAT, and APX increased by 39.2%, 51.3%, 32.3%, and 73.1 percent, respectively, when drought stress was present. On the other hand, biochar lowered antioxidant activities in both irrigation regimes; specifically, SOD, POD, CAT, and APX all saw reductions of 16.0 percent, 23.1 percent, 18.6 percent, and 15.9 percent, respectively. Among the SRNF treatments, the one with the most antioxidant activity was zinc-coated urea (ZCU), while the one without coating was non-coated simple urea (SU) (**Figure 6**). In response to changes in water availability, these results show that biochar and ZCU play a role in controlling sunflower oxidative stress responses.

**Figure 7:**
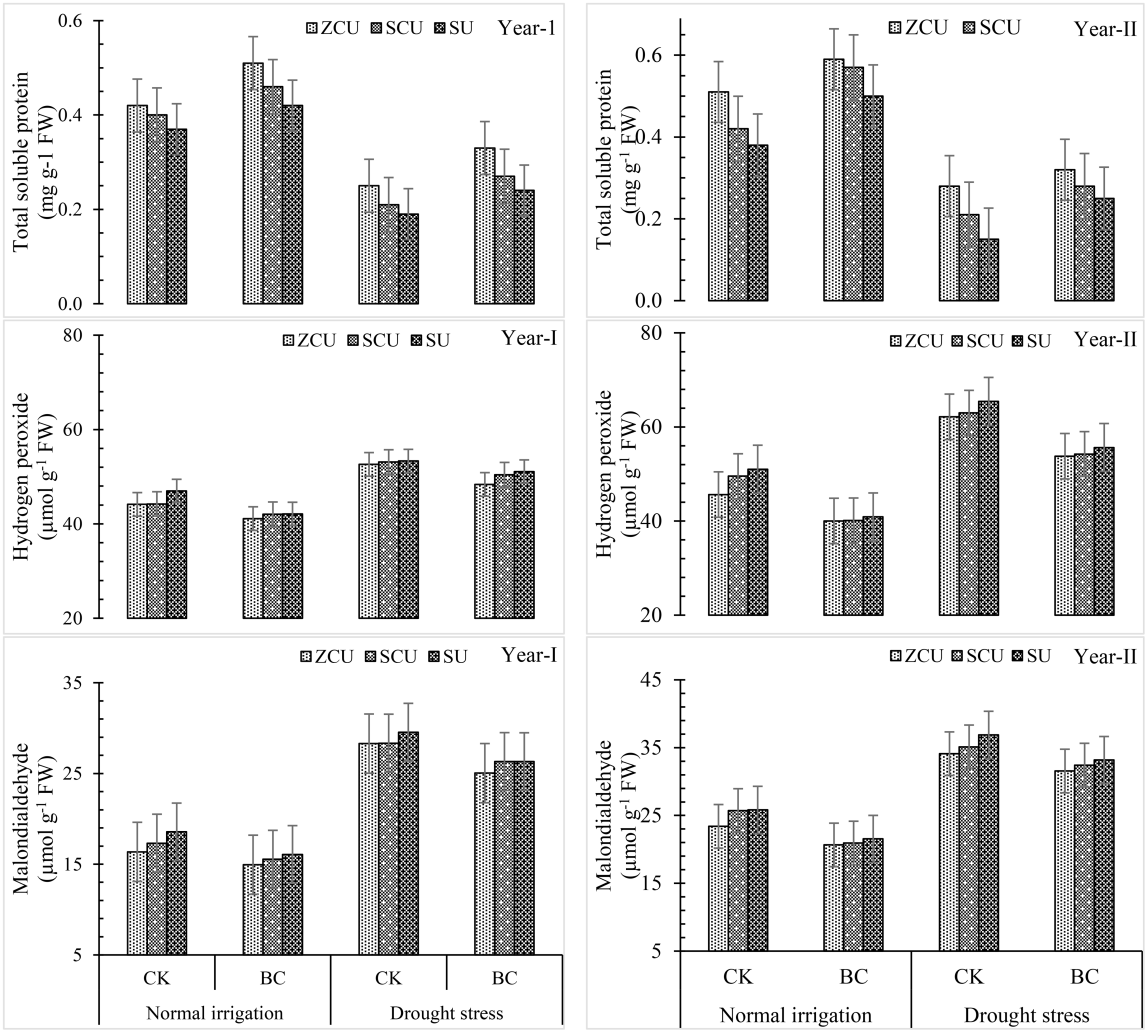
Total soluble protein (TSP), hydrogen peroxide (H_2_O_2_), and malonaldehyde (MDA) effected by slow release nitrogen fertilizers and biochar under drought stress.

Biochar treatments, SRNF applications, and drought stress all led to noticeable changes in total soluble protein (TSP), hydrogen peroxide (H_2_O_2_), and malonaldehyde (MDA) levels. Depending on the severity of the drought, levels of TSP, H_2_O_2_, and MDA could rise as much as 36.1%, 79.7%, and 79.7%, respectively. Under normal irrigation stress, biochar application reduced these stress markers by 19.9%, while under drought stress, it was reduced by 73.1 percent. The levels of H_k_O_k_, APX, and MDA were consistently highest in SU among the SRNF treatments, and consistently lowest in ZCU (**Figure 7**). Both irrigation regimes were able to reduce oxidative stress markers and keep physiological stability intact, thanks to the mitigating effect of biochar and ZCU.

**Figure 8:**
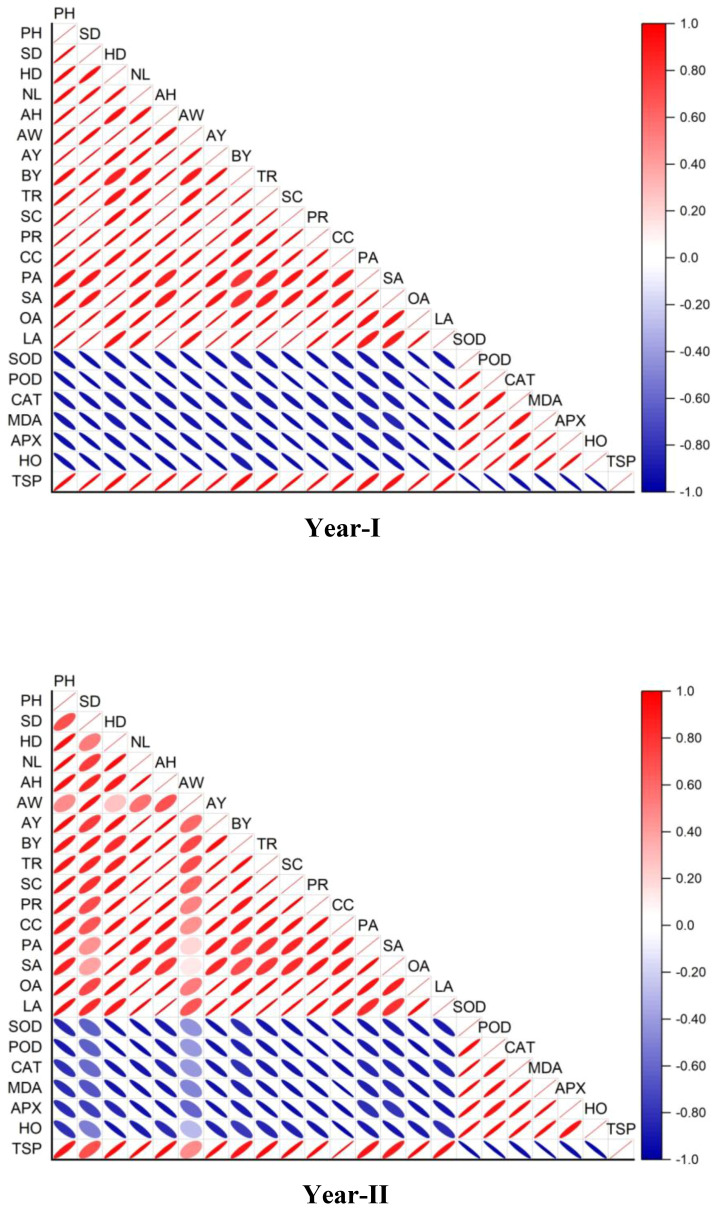
Correlation between the morpho-physiological, antioxidant and biochemical analysis.

The use of SRNF, treatment with biochar, and irrigation schedules all had notable impacts on quality indicators like oil and protein content. The oil and protein contents were highest in ZCU, then in SCU, and finally in SU. The quality parameters were negatively affected by drought stress; however, when biochar was applied, the oil and protein contents were improved in comparison to treatments that did not include biochar (**Figure 8**). Even when sunflower seeds are subjected to drought stress, these results demonstrate that ZCU and biochar improve their quality.

### Correlation

The correlation graph given in **Figure 8** highlights strong positive relationships between growth and yield parameters, indicating the effectiveness of biochar and ZCU in enhancing plant productivity. Negative correlations with stress markers like SOD, POD, and MDA suggest reduced oxidative stress, demonstrating the treatments’ role in mitigating drought effects.

## Discussion

### Drought stress

Our results show that morpho-physiological traits are drastically reduced by drought stress, which also triggers the highest antioxidant activity. Drought stress has emerged as a major threat to agricultural productivity in the face of ongoing global climate change (Sein and Zhi, 2016). Forecasts show that by the century’s end, both the frequency and severity of droughts will have increased (Gutierrez et al., 2014; Sun et al., 2024). Drought has long been known to stunt plant development and interfere with many biochemical and physiological processes. Numerous physiological pathways associated with drought resistance in plants have been the subject of substantial investigation (Zwicke et al., 2015). In order for plants to grow and develop, drought stress interrupts vital internal processes (Lei and Shao, 2023). By contrasting normal irrigation conditions (100 percent field capacity) with drought stress conditions (60 percent field capacity), this study sought to investigate the effects of drought stress on morpho-physiological, biochemical, and growth parameters.

Environmental factors have a significant impact on crop development, yield parameters, and the reactions of plant traits. There was a marked decrease in plant height, stem diameter, head diameter, leaf number, achenes per head, achene yield, and biological yield as a consequence of drought stress, according to the results. Battaglia et al. (2018) found that water scarcity affected maize yield and quality adversely; our findings are in line with theirs. Net photosynthesis (Pn), transpiration rate (E), stomatal conductance (gs), and chlorophyll content (SPAD value) were all found to significantly decrease under drought stress in the present study. In a similar vein, Sekhar et al. (2017) found that drought significantly reduced photosynthesis in mulberry genotypes. Lower leaf water potential, which increases resistance to CO_2_ diffusion, is probably the cause of reduced photosynthetic activity under drought stress (Pflug et al., 2018). Reduced CO_2_ uptake during stomatal closure due to water stress inhibits photosynthesis (Saeidi and Abdoli, 2015). Researchers have found that stomatal conductance drops significantly in drought-stricken wheat genotypes and maize seedlings (Ouyang et al., 2017). All things considered, these results show that drought stress is bad for plants and their ability to produce food.

Hydrogen peroxide (H_2_O_2_) and superoxide anions are reactive oxygen species (ROS) that plants produce in excess when water is not present. These anions impede plant growth and development (Lei and Shao, 2023). Oxidative stress causes proteins, membranes, and enzymes to be damaged when drought stress increases the production of reactive oxygen species (ROS) (Zlatev and Lidon, 2012). Enzymes called catalase (CAT) play a crucial role in reducing oxidative stress and improving plant tolerance to drought by breaking down hydroxide ions into water and oxygen (Hafez et al., 2020). Catalase, superoxide dismutase (SOD), peroxidase (POD), and ascorbate peroxidase are some of the antioxidant defenses that plants activate in response to ROS-induced damage (APX). In stress tolerance, APX is especially important because it converts H_2_O_2_ into water, which protects cells from ROS toxicity and keeps them intact (Kausar et al., 2013; Gharibi et al., 2016). To maintain plant resilience in the face of drought stress, these antioxidant mechanisms are crucial.

### Biochar application

It was noted that one of the possibilities to minimize the harm of drought stress is biochar, or BC (Siebielec et al., 2020; Hafez et al., 2020). Therefore, a substance that can lower DS, BC has received the title of the ‘black gold of agriculture’ Zhang et al., 2019; Khan et al., 2022). This study revealed that the application of BC to crops during the period of drought significantly increased their physiology and antioxidant activity. Licht and Smith (2018) identified that in water-stressed plants, treatment through BC improved photosynthesis rate, transpiration rate, stomatal conductance, and chlorophyll content in plants (Ahmed et al., 2016; Khan et al., 2021). In DS environments BC enhances functionality of the plant through the production of chemicals required for growth (Manolikaki and Diamadopoulos, 2019; Wang et al., 2021).

Some earlier studies Lyu et al. (2016) and Abideen et al. (2020) revealed that BC enhanced the water balance of the plants and enhanced the antioxidant potential. Proline – stress-induced osmolyte being requisite to well regulate osmotic pressure and reduce ROS. According to the present study carried out on Medicago ciliaris Yildirim et al. (2021) found that it has low levels of proline because with BC BC-treated plants had lesser ROS and they were under lesser oxidative and osmotic stress even though DS has potential to increase the proline accumulation substantially. BC shields the photosynthetic apparatus from being damaged by DS due to controlling of electron transport rules and antioxidant intensity (Chaves et al., 2009). In the present study and as stated earlier by Foyer et al. (2009) and Zulfiqar et al. (2022) reported that application of BC enhanced plant metabolic processes, growth, and ROS quenching under DS through enhancing the antioxidant enzymes such as SOD POD CAT and APX.

Arbuscular mycorrhizal fungi (AMF) and BC together have validated potential in improving drought tolerance via osmotic adjustments, hormone regulation, and increased antioxidant activity, among other pathways (Mickan et al., 2016; Hafez et al., 2020). Lower levels of malondialdehyde (MDA), an indicator of reduced lipid peroxidation, were observed after BC application in Medicago ciliaris under DS conditions. Treatment of Brassica oleracea with BC also increased the activity of antioxidant enzymes, which decreased the accumulation of malondialdehyde (Yildirim et al., 2021). According to Khan et al. (2021), BC reduces DS by reducing oxidative and osmotic stress, as evidenced by the fact that it lowers proline and total soluble protein (TSP) levels. The importance of BC in improving physiological processes and bolstering antioxidant defenses in plants makes its impact on drought stress resilience clear in these results.

### Slow releasing nitrogen fertilizers

Results showed that SRNF significantly affected sunflower yield in addition to other physiological and biochemical variables. It was found that zinc-coated urea (ZCU) was the most effective treatment, surpassing sulfur-coated urea (SCU), which had previously beaten non-coated simple urea (SU). The increased yields seen when using ZCU are because of its ability to release nitrogen slowly, increase zinc uptake, and improve agronomic efficiency. Evidence from earlier research shows that bioactive zinc-coated urea improves rice morphology, yield, and quality (Nazir et al., 2021; Shah et al., 2023). The use of slow-release fertilizers greatly enhances crop growth and development, especially in wheat, according to studies conducted by Versino et al. (2020) and Ma et al. (2022). These studies were conducted under field conditions.

Coated fertilizers, such as ZCU, are more effective than their uncoated counterparts because they contain nutrient-solubilizing microorganisms, which allow for a controlled and sustained release of nitrogen (Shah et al., 2023). By dissolving insoluble fractions in the soil rhizosphere, the bioactive compounds in ZCU increase nutrient bioavailability and guarantee a consistent supply of vital nutrients. Enzyme activation, protein synthesis, nucleic acid and starch metabolism, and zinc’s specific function in these areas are all essential for vital processes like pollination (Cakmak and Kutman, 2018). Coated fertilizers increase the soil’s inorganic nitrogen availability while conventional urea quickly hydrolyzes, resulting in substantial nitrogen losses.

In addition, when compared to regular urea, ZCU was superior in improving chlorophyll pigments. Nitrogen is crucial for the absorption of light energy in photosynthesis because it is a component of chlorophyll (Earl and Tollenaar, 1997). Consistent with earlier research, our results demonstrate that coated urea enhances photosynthetic pigment synthesis. As an illustration, Scharf et al. (2006) found that seedlings treated with coated urea had a significantly higher chlorophyll content. Consistent with prior research, this study found that increased yields were associated with growth-related traits and more efficient nitrogen use (Otteson et al., 2007; Zheng et al., 2016; Haroon et al., 2023; Anjum et al., 2022). Coated urea improves crop performance, according to these studies.

The findings of this study align with previous research indicating that the application of slow-release nitrogen fertilizers (SRNF), particularly zinc-coated urea (ZCU), and biochar significantly enhance the fatty acid composition of sunflower seeds under both normal and drought stress conditions. Nazir et al. (2021) reported that the use of bio-activated zinc oxide (ZnO) coated urea, a form of SRNF, resulted in a 15–20% improvement in yield and biochemical parameters of rice crops, which is comparable to the observed increases in palmitic (18.8–19.9%), stearic (18.4–25.0%), oleic (16.1–19.9%), and linoleic (20.1–21.0%) acid levels in our study. Similarly, Khan et al. (2022) found that ZCU application led to a 94% increase in dry matter yield and a 75% enhancement in nitrogen uptake in ryegrass, supporting our findings on improved nutrient uptake efficiency contributing to better oil quality. The effectiveness of SRNF in improving oil composition is attributed to its gradual nitrogen release, ensuring sustained nutrient availability during critical growth stages, thus improving biochemical pathways responsible for lipid synthesis. The role of biochar in enhancing soil water retention and nutrient availability, as reported by Mandal et al. (2020), further corroborates our results, where biochar application increased fatty acid levels by 37.2%, 34.6%, 11.3%, and 9.40% for palmitic, stearic, oleic, and linoleic acids, respectively, under normal irrigation conditions. Under drought stress, the effectiveness of biochar was also evident, with increases of 28.9%, 29.5%, 10.7%, and 7.71%, respectively. These findings suggest that integrating biochar with SRNF, especially ZCU, not only enhances the fatty acid profile but also mitigates the adverse effects of drought stress, making sunflower cultivation more resilient and sustainable.

## Conclusion

Finally, when it came to improving morpho-physiological performance, zinc-coated urea, sulfur-coated urea, and non-coated simple urea were the most effective. The use of biochar improved morpho-physiological traits in both normal and drought-stressed environments and considerably increased antioxidant activity during drought stress. So, to maximize yield and improve overall crop performance, a promising strategy for sustainable sunflower productivity is to combine biochar with slow-release nitrogen fertilizers. The findings further emphasize the potential of biochar and ZCU in creating a synergistic effect that enhances crop tolerance to abiotic stressors like drought. This combination not only ensures improved water use efficiency but also sustains the nutrient availability required for optimal plant growth and yield. Moreover, the study provides a valuable framework for adopting sustainable agricultural practices that promote environmental conservation and resilience in crop production systems. Such integrated strategies are crucial for addressing the dual challenges of increasing food demand and mitigating the adverse impacts of climate change.

## Data Availability

The original contributions presented in the study are included in the article/supplementary material, further inquiries can be directed to the corresponding author/s.

## References

[B1] Abideen Z. Koyro H. W. Huchzermeyer B. Bilquees G. U. L. Khan M. A. (2020). Impact of a biochar or a biochar-compost mixture on water relation, nutrient uptake and photosynthesis of Phragmites karka. Pedosphere 30, 466–477. doi: 10.1016/S1002-0160(17)60362-X

[B2] Agarwal P. Parida S. K. Raghuvanshi S. Kapoor S. Khurana P. Khurana J. P.. (2016). Rice improvement through genome-based functional analysis and molecular breeding in India. Rice 9, 1–17. doi: 10.1186/s12284-015-0073-2 26743769 PMC4705060

[B3] Ahmed F. Arthur E. Plauborg F. Andersen M. N. (2016). Biochar effects on maize physiology and water capacity of sandy subsoil. Mechanization Agric. Conserving Resour. 62, 8–13.

[B4] Anjum L. Rehman A. Rizwan M. Hussain S. Waqas M. S. (2022). Impact of integrated nutrient management on yield of different varieties of oat. Environ. Sci. Proc. 23, 14. doi: 10.3390/environsciproc2022023014

[B5] Battaglia M. L. Lee C. Thomason W. (2018). Corn yield components and yield responses to defoliation at different row widths. Agron. J. 110, 210–225. doi: 10.2134/agronj2017.06.0322

[B6] Cakmak I. Kutman U.Á. (2018). Agronomic biofortification of cereals with zinc: a review. Eur. J. Soil Sci. 69, 172–180. doi: 10.1111/ejss.2018.69.issue-1

[B7] Chance B. Maehly A. C. (1955). Assay of catalases and peroxidases. Methods Enzymol. 2, 764–775. doi: 10.1016/S0076-6879(55)02300-8 13193536

[B8] Chaves M. M. Flexas J. Pinheiro C. (2009). Photosynthesis under drought and salt stress: regulation mechanisms from whole plant to cell. Ann. Bot. 103, 551–560. doi: 10.1093/aob/mcn125 18662937 PMC2707345

[B9] Chen J. Y. Miao Y. Sato S. Zhang H. (2008). Near-infrared spectroscopy for determination of the protein composition of rice flour. Food Sci. Technol. Res. 14, 132–138. doi: 10.3136/fstr.14.132

[B10] Cui G. Zhao X. Liu S. Sun F. Zhang C. Xi Y. (2017). Beneficial effects of melatonin in overcoming drought stress in wheat seedlings. Plant Physiol. Biochem. 118, 138–149. doi: 10.1016/j.plaphy.2017.06.014 28633086

[B11] Earl H. J. Tollenaar M. (1997). Maize leaf absorptance of photosynthetically active radiation and its estimation using a chlorophyll meter. Crop Sci. 37, 436–440. doi: 10.2135/cropsci1997.0011183X003700020022x

[B12] Ebrahimian E. Seyyedi S. M. Bybordi A. Damalas C. A. (2019). Seed yield and oil quality of sunflower, safflower, and sesame under different levels of irrigation water availability. Agric. Water Manage. 218, 149–157. doi: 10.1016/j.agwat.2019.03.031

[B13] Foyer C. H. Bloom A. J. Queval G. Noctor G. (2009). Photorespiratory metabolism: genes, mutants, energetics, and redox signaling. Annu. Rev. Plant Biol. 60, 455–484. doi: 10.1146/annurev.arplant.043008.091948 19575589

[B14] Gharibi S. Tabatabaei B. E. S. Saeidi G. Goli S. A. H. (2016). Effect of drought stress on total phenolic, lipid peroxidation, and antioxidant activity of Achillea species. Appl. Biochem. Biotechnol. 178, 796–809. doi: 10.1007/s12010-015-1909-3 26541161

[B15] Giannopolitis C. N. Ries S. K. (1977). Superoxide dismutases: I. Occurrence in higher plants. Plant Physiol. 59, 309–314. doi: 10.1104/pp.59.2.309 16659839 PMC542387

[B16] Gutierrez F. Parise M. De Waele J. Jourde H. (2014). A review on natural and human-induced geohazards and impacts in karst. Earth-Science Rev. 138, 61–88. doi: 10.1016/j.earscirev.2014.08.002

[B17] Hafez Y. Attia K. Alamery S. Ghazy A. Al-Doss A. Ibrahim E.. (2020). Beneficial effects of biochar and chitosan on antioxidative capacity, osmolytes accumulation, and anatomical characters of water-stressed barley plants. Agronomy 10, 630. doi: 10.3390/agronomy10050630

[B18] Haider I. Raza M. A. S. Iqbal R. Aslam M. U. Habib-ur-Rahman M. Raja S.. (2020). Potential effects of biochar application on mitigating the drought stress implications on wheat (Triticum aestivum L.) under various growth stages. J. Saudi Chem. Soc. 24, 974–981. doi: 10.1016/j.jscs.2020.10.005

[B19] Haroon Z. Cheema M. J. M. Saleem S. Amin M. Anjum M. N. Tahir M. N.. (2023). Potential of precise fertilization through adoption of management zones strategy to enhance wheat production. Land 12, 540. doi: 10.3390/land12030540

[B20] Heath R. L. Packer L. (1968). Photoperoxidation in isolated chloroplasts: I. Kinetics and stoichiometry of fatty acid peroxidation. Arch. Biochem. Biophysics 125, 189–198. doi: 10.1016/0003-9861(68)90654-1 5655425

[B21] Hochman Z. Horan H. (2018). Causes of wheat yield gaps and opportunities to advance the water-limited yield frontier in Australia. Field Crops Res. 228, 20–30. doi: 10.1016/j.fcr.2018.08.023

[B22] Kausar R. Arshad M. Shahzad A. Komatsu S. (2013). Proteomics analysis of sensitive and tolerant barley genotypes under drought stress. Amino Acids 44, 345–359. doi: 10.1007/s00726-012-1338-3 22707152

[B23] Khan Z. Khan M. N. Zhang K. Luo T. Zhu K. Hu L. (2021). The application of biochar alleviated the adverse effects of drought on the growth, physiology, yield and quality of rapeseed through regulation of soil status and nutrients availability. Ind. Crops Products 171, 113878. doi: 10.1016/j.indcrop.2021.113878

[B24] Khan W. U. D. Shaukat R. Farooq M. A. Ashraf M. N. Nadeem F. Tanveer M.. (2022). Iron-doped biochar regulated soil nickel adsorption, wheat growth, its physiology and elemental concentration under contrasting abiotic stresses. Sustainability 14, 7852. doi: 10.3390/su14137852

[B25] Lei L. Shao H. (2023). Plant growth stimulatory effect of terrein and its mechanism of action in crops under drought stress. Agriculture 13, 1889. doi: 10.3390/agriculture13101889

[B26] Licht J. Smith N. (2018). The influence of lignocellulose and hemicellulose biochar on photosynthesis and water use efficiency in seedlings from a Northeastern US pine-oak ecosystem. J. Sustain. Forestry 37, 25–37. doi: 10.1080/10549811.2017.1386113

[B27] Lowry O. H. Rosebrough N. J. Farr A. L. Randall R. J. (1951). Protein measurement with the Folin phenol reagent. J. Biol. Chem. 193, 265–275. doi: 10.1016/S0021-9258(19)52451-6 14907713

[B28] Lyu S. Du G. Liu Z. Zhao L. Lyu D. (2016). Effects of biochar on photosystem function and activities of protective enzymes in Pyrus ussuriensis Maxim. under drought stress. Acta Physiologiae Plantarum 38, 1–10. doi: 10.1007/s11738-016-2236-1

[B29] Ma Q. Tao R. Ding Y. Zhang X. Li F. Zhu M.. (2022). Can split application of slow-release fertilizer improve wheat yield, nitrogen efficiency and their stability in different ecological regions? Agronomy 12, 407. doi: 10.3390/agronomy12020407

[B30] Mandal S. Pu S. He L. Ma H. Hou D. (2020). Biochar induced modification of graphene oxide & nZVI and its impact on immobilization of toxic copper in soil. Environ. pollut. 259, 113851. doi: 10.1016/j.envpol.2019.113851 31918134

[B31] Manolikaki I. Diamadopoulos E. (2019). Positive effects of biochar and biochar-compost on maize growth and nutrient availability in two agricultural soils. Commun. Soil Sci. Plant Anal. 50, 512–526. doi: 10.1080/00103624.2019.1566468

[B32] Mickan B. S. Abbott L. K. Stefanova K. Solaiman Z. M. (2016). Interactions between biochar and mycorrhizal fungi in a water-stressed agricultural soil. Mycorrhiza 26, 565–574. doi: 10.1007/s00572-016-0693-4 27067713

[B33] Naz M. Y. Sulaiman S. A. (2016). Slow release coating remedy for nitrogen loss from conventional urea: a review. J. Controlled Release 225, 109–120. doi: 10.1016/j.jconrel.2016.01.037 26809006

[B34] Nazir Q. Wang X. Hussain A. Ditta A. Aimen A. Saleem I.. (2021). Variation in growth, physiology, yield, and quality of wheat under the application of different zinc-coated formulations. Appl. Sci. 11, 4797. doi: 10.3390/app11114797

[B35] Otteson B. N. Mergoum M. Ransom J. K. (2007). Seeding rate and nitrogen management effects on spring wheat yield and yield components. Agron. J. 99, 1615–1621. doi: 10.2134/agronj2007.0002

[B36] Ouyang W. Struik P. C. Yin X. Yang J. (2017). Stomatal conductance, mesophyll conductance, and transpiration efficiency in relation to leaf anatomy in rice and wheat genotypes under drought. J. Exp. Bot. 68, 5191–5205. doi: 10.1093/jxb/erx314 28992130 PMC5853379

[B37] Paneque M. José M. Franco-Navarro J. D. Colmenero-Flores J. M. Knicker H. (2016). Effect of biochar amendment on morphology, productivity, and water relations of sunflower plants under non-irrigation conditions. Catena 147, 280–287. doi: 10.1016/j.catena.2016.07.037

[B38] Pflug E. E. Buchmann N. Siegwolf R. T. Schaub M. Rigling A. Arend M. (2018). Resilient leaf physiological response of European beech (Fagus sylvatica L.) to summer drought and drought release. Front. Plant Sci. 9, 187. doi: 10.3389/fpls.2018.00187 29515605 PMC5825912

[B39] Poiroux-Gonord F. Santini J. Fanciullino A. L. Lopez-Lauri F. Giannettini J. Sallanon H.. (2013). Metabolism in orange fruits is driven by photooxidative stress in the leaves. Physiologia Plantarum 149, 175–187. doi: 10.1111/ppl.2013.149.issue-2 23330573

[B40] Qayyum M. F. Haider G. Iqbal M. Hameed S. Ahmad N. Rehman M. Z. U.. (2021). Effect of alkaline and chemically engineered biochar on soil properties and phosphorus bioavailability in maize. Chemosphere 266, 128980. doi: 10.1016/j.chemosphere.2020.128980 33243575

[B41] Rahman M. H. Ahmad A. Wang X. Wajid A. Nasim W. Hussain M.. (2018). Multi-model projections of future climate and climate change impacts uncertainty assessment for cotton production in Pakistan. Agric. For. Meteorology 253, 94–113. doi: 10.1016/j.agrformet.2018.02.008

[B42] Ramzani P. M. A. Shan L. Anjum S. Ronggui H. Iqbal M. Virk Z. A.. (2017). Improved quinoa growth, physiological response, and seed nutritional quality in three soils having different stresses by the application of acidified biochar and compost. Plant Physiol. Biochem. 116, 127–138. doi: 10.1016/j.plaphy.2017.05.003 28554146

[B43] Saeidi M. Abdoli M. (2015). Effect of drought stress during grain filling on yield and its components, gas exchange variables, and some physiological traits of wheat cultivars. J. Agric. Sci. Technol. 17, 885–898.

[B44] Scharf P. C. Brouder S. M. Hoeft R. G. (2006). Chlorophyll meter readings can predict nitrogen need and yield response of corn in the north-central USA. Agron. J. 98, 655–665. doi: 10.2134/agronj2005.0070

[B45] Sein Z. M. M. Zhi X. (2016). Interannual variability of summer monsoon rainfall over Myanmar. Arabian J. Geosciences 9, 1–19. doi: 10.1007/s12517-016-2502-y

[B46] Sekhar K. M. Reddy K. S. Reddy A. R. (2017). Amelioration of drought-induced negative responses by elevated CO_2_ in field-grown short rotation coppice mulberry (Morus *spp.*), a potential bio-energy tree crop. Photosynthesis Res. 132, 151–164. doi: 10.1007/s11120-017-0351-5 28238122

[B47] Shah M. N. Wright D. L. Hussain S. Koutroubas S. D. Seepaul R. George S.. (2023). Organic fertilizer sources improve the yield and quality attributes of maize (Zea mays L.) hybrids by improving soil properties and nutrient uptake under drought stress. J. King Saud University-Science 35, 102570. doi: 10.1016/j.jksus.2023.102570

[B48] Siebielec S. Siebielec G. Klimkowicz-Pawlas A. Gałązka A. Grządziel J. Stuczyński T. (2020). Impact of water stress on microbial community and activity in sandy and loamy soils. Agronomy 10, 1429. doi: 10.3390/agronomy10091429

[B49] Sun M. Li X. Xu H. Wang K. Anniwaer N. Hong S. (2024). Drought thresholds that impact vegetation reveal the divergent responses of vegetation growth to drought across China. Global Change Biol. 30, e16998. doi: 10.1111/gcb.16998 37899690

[B50] Sunaina B. Kumar J. R. Rupak K. Mahesh R. (2019). A case study on soil fertility status and maize productivity in Dang District, Nepal. Malaysian J. Sustain. Agric. (MJSA) 3, 56–59. doi: 10.26480/mjsa.02.2019.56.59

[B51] Tang H. Wang S. Liu Y. Hassan M. U. Song Y. Huang G.. (2022). Biochar: A promising soil amendment to mitigate heavy metals toxicity in plants. Notulae Botanicae Horti Agrobotanici Cluj-Napoca 50, 12778. doi: 10.15835/nbha50312778

[B52] Velikova V. Yordanov I. Edreva A. (2000). Oxidative stress and some antioxidant systems in acid rain-treated bean plants: protective role of exogenous polyamines. Plant Sci. 151, 59–66. doi: 10.1016/S0168-9452(99)00197-1

[B53] Versino F. Urriza M. García M. A. (2020). Cassava-based biocomposites as fertilizer controlled-release systems for plant growth improvement. Ind. Crops Products 144, 112062. doi: 10.1016/j.indcrop.2019.112062

[B54] Vijayaraghavareddy P. Lekshmy S. V. Struik P. C. Makarla U. Yin X. Sreeman S. (2022). Production and scavenging of reactive oxygen species confer to differential sensitivity of rice and wheat to drought stress. Crop Environ. 1, 15–23. doi: 10.1016/j.crope.2022.03.010

[B55] Wang S. Zheng J. Wang Y. Yang Q. Chen T. Chen Y.. (2021). Photosynthesis, chlorophyll fluorescence, and yield of peanut in response to biochar application. Front. Plant Sci. 12, 650432. doi: 10.3389/fpls.2021.650432 34135920 PMC8200678

[B56] Yildirim E. Ekinci M. Turan M. (2021). Impact of biochar in mitigating the negative effect of drought stress on cabbage seedlings. J. Soil Sci. Plant Nutr. 21, 2297–2309. doi: 10.1007/s42729-021-00522-z

[B57] Zhang F. F. Gao S. Zhao Y. Y. Zhao X. L. Liu X. M. Xiao K. (2015). Growth traits and nitrogen assimilation-associated physiological parameters of wheat (Triticum aestivum L.) under low and high N conditions. J. Integr. Agric. 14, 1295–1308. doi: 10.1016/S2095-3119(14)60957-6

[B58] Zhang M. Riaz M. Zhang L. El-Desouki Z. Jiang C. (2019). Biochar induces changes to basic soil properties and bacterial communities of different soils to varying degrees at 25 mm rainfall: more effective on acidic soils. Front. Microbiol. 10, 1321. doi: 10.3389/fmicb.2019.01321 31249563 PMC6582450

[B59] Zheng W. Zhang M. Liu Z. Zhou H. Lu H. Zhang W.. (2016). Combining controlled-release urea and normal urea to improve the nitrogen use efficiency and yield under wheat-maize double cropping system. Field Crops Res. 197, 52–62. doi: 10.1016/j.fcr.2016.08.004

[B60] Zlatev Z. Lidon F. C. (2012). An overview on drought induced changes in plant growth, water relations and photosynthesis. Emirates J. Food Agric. (EJFA) 24, 1.

[B61] Zulfiqar B. Raza M. A. S. Saleem M. F. Aslam M. U. Iqbal R. Muhammad F.. (2022). Biochar enhances wheat crop productivity by mitigating the effects of drought: Insights into physiological and antioxidant defense mechanisms. PloS One 17, e0267819. doi: 10.1371/journal.pone.0267819 35482811 PMC9049366

[B62] Zwicke M. Picon-Cochard C. Morvan-Bertrand A. Prud’homme M. P. Volaire F. (2015). What functional strategies drive drought survival and recovery of perennial species from upland grassland? Ann. Bot. 116, 1001–1015. doi: 10.1093/aob/mcv037 25851134 PMC4640119

